# Impact of the Mediterranean Diet on stroke incidence and cognitive impairment in CADASIL and CAA patients: the DIETETICA study

**DOI:** 10.3389/fnut.2025.1682134

**Published:** 2025-11-25

**Authors:** Camilla Strazzabosco, Benedetta Storti, Giulia Marinoni, De Toma Carolina, Isabella Canavero, Nicola Rifino, Giorgio Boncoraglio, Esteban Zacarias, Alessandro Francia, Giuliana Pollaci, Tatiana Carrozzini, Antonella Potenza, Gemma Gorla, Ramona De Amicis, Laura Gatti, Anna Bersano

**Affiliations:** 1 Cerebrovascular Unit, Fondazione I.R.C.C.S. Istituto Neurologico "Carlo Besta", Milan, Italy; 2 Department of Biomedical and Clinical Sciences, University of Milan, Milan, Italy; 3 International Center for the Assessment of Nutritional Status and the Development of Dietary Intervention Strategies (ICANS-DIS), Department of Food, Environmental and Nutritional Sciences (DeFENS), University of Milan, Milan, Italy; 4 IRCCS Istituto Auxologico Italiano, Obesity Unit and Laboratory of Nutrition and Obesity Research, Department of Endocrine and Metabolic Diseases, Milan, Italy

**Keywords:** cerebral small vessel disease, CADASIL, CAA, Mediterranean Diet, nutritional assessments, nutritional adequacy ratio

## Abstract

**Introduction:**

Cerebral small vessel disease (cSVD), including Cerebral Autosomal Dominant Arteriopathy with Subcortical Infarcts and Leukoencephalopathy (CADASIL) and Cerebral Amyloid Angiopathy (CAA), is a major cause of non-sporadic stroke and cognitive impairment. Despite the recognized benefits of the Mediterranean Diet (MD) on cardiovascular and neurodegenerative diseases, its role in cSVD has not been yet investigated.

**Methods:**

The DIETETICA study (“Effect of Mediterranean Diet on stroke incidence and cognitive impairment in patients with CADASIL and CAA”) aims to explore the association between MD adherence, stroke incidence and cognitive impairment in patients affected by CADASIL and CAA. During observational phase, 22 participants were recruited: 9 with CADASIL and 13 with CAA. 68.18% were male and 31.82% were female.

**Results:**

According to the response obtained through a validated 14-item questionnaire, 63.63% of the total cohort had moderate adherence to MD, 27.27% had low adherence, and 9.09% had high adherence. Differences in body composition, in terms of fat mass (FM) and fat-free mass (FFM), were observed between the two patient cohorts. Overall, the preliminary observations regarding dietary daily intake were characterized by inadequate of nutrients which are known to have neurovascular benefits, such as polyunsaturated fatty acids (PUFAs), calcium, potassium and vitamin D, for both CADASIL and CAA patients.

**Conclusion:**

The present study will pave the way to the evaluation of the effects of MD pattern on cSVD, in order to provide evidence-based dietary recommendations aimed at reducing stroke incidence and cognitive impairment.

## Introduction

Cerebral small vessel disease (cSVD) represents a cluster of disorders affecting the small arteries, arterioles, venules, and capillaries of the brain, and refers to several pathological processes and etiologies (1). It is an important cause of stroke (25% of strokes are lacunar) and intracerebral hemorrhage. Rare heritable diseases have been identified as potential causes of cSVD, with Cerebral Autosomal Dominant Arteriopathy with Subcortical Infarcts and Leukoencephalopathy (CADASIL) being the most common form. It is an autosomal dominant disorder caused by pathogenic variants in the NOTCH3 gene on chromosome 19 (2). CADASIL is characterized by alterations in small penetrating arteries, arterioles, and capillaries in the brain, leading to a non-atherosclerotic angiopathy that primarily affects the central nervous system; it manifests with migraine with aura, subcortical ischemic stroke, progressive cognitive impairment and mood disorders (3). However, age-related and hypertension-associated cSVD, along with Cerebral Amyloid Angiopathy (CAA), represent the most prevalent forms of the disease (4). Particularly, CAA is an emerging not heritable cSVD characterized by the accumulation of amyloid fibrils in the walls of small-to medium-sized arterial blood vessels and in capillaries of the central nervous parenchyma and leptomeninges. CAA is a significant cause of lobar intracerebral hemorrhages (ICH), progressive cognitive impairment in elderly patients and, transient neurological episodes (TFNEs) (5–7). Although CADASIL and CAA differ in their underlying pathophysiology and histopathological characteristics, they share common clinical and molecular features: (1) both represent major etiologies of cSVD, with CADASIL being hereditary and CAA acquired (2, 7); (2) they are associated with similar clinical manifestations, including an increased risk of stroke, cognitive impairment, and intracerebral hemorrhage (8); (3) both conditions show abnormal accumulation of proteins within the vessel wall (7, 9); and (4) they display overlapping profiles in terms of protein expression and affected vascular cell types (10).

Ischemic and hemorrhagic strokes, the main clinical manifestations of CADASIL and CAA, respectively (2, 7), can contribute to malnutrition through a range of mechanisms, including motor deficits, cognitive impairment, altered consciousness, neurogenic vomiting, dysphagia, depression, and gastrointestinal dysfunction (11). Additionally, stroke patients are prone to secondary complications such as bone mineral density loss and sarcopenia (12).

Currently, there are no specific treatments available for either CADASIL or CAA. Clinical management is primarily aimed at preventing further cerebrovascular events by controlling conventional vascular risk factors such as hypertension, dyslipidemia and smoking (6, 13).

The Mediterranean Diet (MD) has been recognized for its beneficial effects in several conditions, including obesity, metabolic syndrome, hypertension, diabetes and cardiovascular disease (14–18). Furthermore, several studies have highlighted a beneficial effect of MD on neurodegenerative diseases, such as Alzheimer’s and Parkinson’s diseases (19) as well as in reducing the incidence of both ischemic and hemorrhagic stroke (20). MD, which was common among Mediterranean populations in the 1950s, is characterized by high consumption of olive oil, fruits, vegetables, legumes, nuts, seeds, and unrefined grains, along with moderate-to-high consumption of fish and low consumption of red meat and sugar-sweetened products. These nutrients are recognized to improve endothelial function (21), a critical factor given that cSVD is fundamentally a disease of the small vessels where endothelial integrity is compromised. Furthermore, they possess anti-inflammatory and antioxidant properties (22, 23) which are crucial in mitigating the chronic inflammatory state associated with cSVD. While recent studies on a general elderly population in Northern Italy (24), have confirmed an association between higher Mediterranean Diet adherence and better cognitive status, to our knowledge, no study has yet specifically investigated this link in high-risk clinical populations such as patients with CADASIL or CAA. The DIETETICA study aims to overcome the current lack of data by:

- *Phase I* (observational): assessing anthropometric parameters, body composition as well as nutrition status in patients with CADASIL and CAA;
- *Phase II* (interventional): evaluating the impact of the MD in patients with CADASIL and CAA, in order to provide evidence-based dietary recommendations for this patient population.

## Patients and methods

### Study design

DIETETICA is a single-center study, structured in two different phases. *Phase I* is an observational phase, conducted between October 2024 and March 2025. It aimed to collect comprehensive patient data, including anthropometric and body composition parameters, daily dietary intake with a particular focus on nutrients typical of the MD, physical activity levels, nutritional status and neuropsychological screening assessment.

*Phase II*, the interventional phase, is scheduled to take place from June 2025 to June 2026. It will consist of a randomized controlled clinical trial involving two intervention groups: one group will follow a MD supplemented with olive oil, while the other will follow a MD supplemented with dried fruits. These intervention groups will be compared to a control group, which, for ethical reasons, will receive education on the benefits of a low-fat diet, given that all participants are at high risk for cerebrovascular events. These intervention groups will be compared to a control group, which, for ethical reasons, will receive education on the benefits of a low-fat diet, given that all participants are at high risk for cerebrovascular events. At the baseline of *phase II*, participants will undergo a re-assessment of anthropometric and body composition parameters, physical activity levels, adherence to the MD, and a more comprehensive neuropsychological evaluation. Follow-up visits will be conducted on-site at 12 months, supplemented by monthly phone calls to support compliance and detect any new cerebrovascular events. To promote adherence, participants in the MD groups will receive education on the benefits of the MD along with a monthly menu including recipes. Participants in the control group will receive education on the benefits of a low-fat diet, with an emphasis on reducing both saturated and unsaturated fats.

For *phase II,* participants will be randomly assigned to one of the intervention groups using a 1:1:1 allocation ratio. Given the nature of the dietary interventions, participants will be aware of their assigned group. To minimize bias, outcome assessors will be blinded to group allocation. This study is registered with ClinicalTrials.gov, NCT06933212.

**Figure 1:**
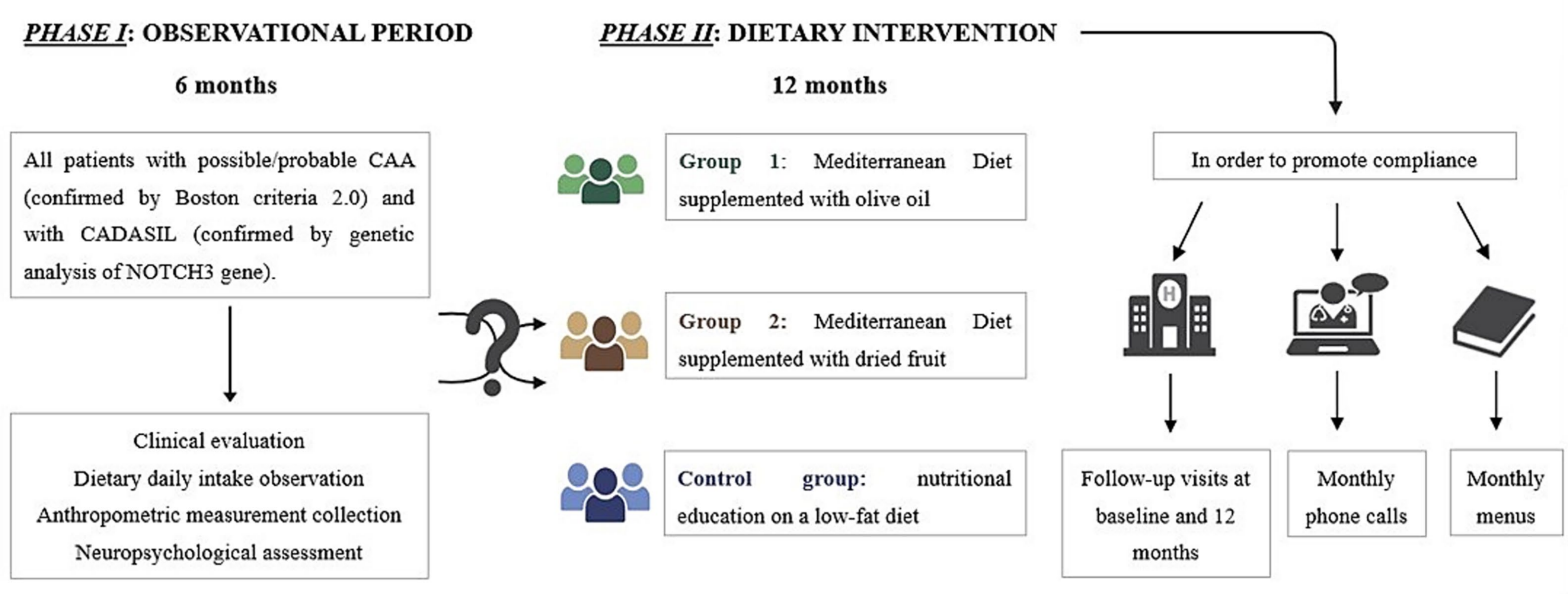
Representative framework of the DIETETICA study design.

A schematic representation of DIETETICA study design is reported in Figure 1.

### Study population

All consecutive patients referring to the Cerebrovascular Diseases Unit at Fondazione I. R. C. C. S. Istituto Neurologico “C. Besta” with a diagnosis of CADASIL (confirmed by genetic analysis of *NOTCH3* gene) and possible/probable CAA (defined according to Boston Criteria 2.0) (25), were recruited.

Inclusion criteria were:

- Age ≥18 years;
- Clinical stability (no acute cerebrovascular events in the previous 3 months);
- Ability and willingness to adhere to a long-term dietary protocol;
- Sufficient functional autonomy to manage daily food preparation or presence of a caregiver able to assist.

Exclusion criteria included:

- Diagnosis of a major neurocognitive disorder, as defined by the Diagnostic and Statistical Manual of Mental Disorders (DSM-5-TR);
- Inability to follow the assigned dietary intervention due to personal, family, or religious reasons, or any other circumstance (e.g., socioeconomic or health-related factors) that could interfere with adherence to the nutritional protocol;
- Participation in another interventional trial or currently following a specific diet.

All participants were required to provide informed consent prior to study enrolment.

## Clinical data collection

### Anthropometric measurements and body composition

Anthropometric measurements are collected according to the conventional criteria and measuring procedures proposed by Lohman (26). All patients undergo the following anthropometric measurements: body weight (BW, kg), body height (BH, cm), body circumference (cm) and skinfold thickness (mm). All the measurements are performed on the non-dominant side of the body.

BW is measured using a digital floor scale (*Seca 877, Seca Corporation*), with an accuracy of 100 g, while participants wear only light underwear and after emptying their bladder. BH is measured closer to 0.1 cm using a vertical stadiometer. Body mass index (BMI) is calculated using the following formula: BMI = BW kg/BH m^2^. According to BMI participants are divided in four categories: underweight (BMI < 18.5 kg/m^2^), normal weight (BMI 18.5–24.9 kg/m^2^), overweight (BMI 25.0–29.9 kg/m^2^) and obesity (BMI > 30.0 kg/ m^2^). Body circumference such as arm circumference (AC), waist circumference (WC), hip circumference (HC) and calf circumference (CC) are taken with a non-stretch tape. AC is measured at the mid-point between the acromion and the olecranon. WC is measured at the mid-point between the lower rib margin and the superior anterior iliac spine to the nearest 0.5 cm. HC and CC are measured at the level of the widest point of the hips and calves, respectively.

Skinfold thickness is measured by GIMA skinfold caliper (*GIMA 27320, Gima S.p. A., Gessate, Italy*) with a sensitivity of 0.2 mm and constant pressure of 10 g/sq. mm. Four skinfolds are taken: biceps, triceps, subscapular, and suprailiac. Each skinfold thickness is measured 3 times and the mean value is calculated. Body density and fat mass (% of BW; FM) are calculated using the Durnin and Womersley method and by Siri formula (27, 28), respectively. Fat mass (FM) is then converted to kilograms, which allow for the estimation of fat-free mass (FFM) in kilograms. This method provides a reliable and practical estimation of body composition in a clinical setting, although it is recognized to have lower precision compared to imaging techniques such as dual-energy X-ray absorptiometry (DEXA). To minimize measurement variability and ensure data consistency, all anthropometric measurements were performed by the same trained nutritionist with a low intra-operator technical error of measurement. This standardized approach enhances the reliability of body composition estimation via the skinfold method within our study.

### Physical activity level and risk of malnutrition

Physical activity level is assessed using the validated 7-item International Physical Activity Questionnaire (IPAQ), which evaluates activity over the past 7 days (29). Activity volume is calculated by weighting each activity type according to its energy expenditure, expressed in metabolic equivalents (METs), and the total daily METs for each patient is determined. Through METs calculations, the physical activity level is estimated, enabling the estimation of total daily energy expenditure (TDEE) using the basal metabolic rate (BMR) determined by Harris-Benedict formula (30).

Mini Nutritional Assessment (MNA) is used to identify malnourished patients or those at risk of malnutrition in elderly individuals in clinics, hospitals and nursing homes (31). MNA is a validated test of 18 multiple choice questions divided into two parts. A final score which indicates the level of the risk of malnutrition (low, medium or high), is obtained. A score between 24 and 30 indicates an adequate nutritional status, 17–23.5 shows a risk of malnutrition and a value < 17 is an indicator of malnutrition or poor nutritional status.

### Nutritional assessment

Dietary data, including all the foods consumed by patients and their respective quantities, are obtained using a 7-day food record (7-dFR). The dietary records are analyzed using *MetaDieta* software (version 4.7.1), which provides the average daily intake of energy, protein, carbohydrates, fiber, total fat, saturated fatty acids (SFAs), monounsaturated fatty acids (MUFAs), polyunsaturated fatty acids (PUFAs), including *ω*-6 and ω-3 PUFAs, as well as eicosapentaenoic acid (EPA) and docosahexaenoic acid (DHA). Additionally, the average daily intake of minerals (calcium, potassium, iron, phosphorus, sodium), fat-soluble vitamins (vitamins A, D, E) and water-soluble vitamins (vitamins C, B9, B12) is also assessed. Nutritional adequacy is evaluated using the Nutritional Adequacy Ratio (NAR), which expresses the individual’s intake of a nutrient as a percentage of the corresponding Italian Dietary Reference Values (DRVs) for that nutrient (32). Specifically, to calculated the NAR, values such as Population Reference Intake (PRI), Suggested Dietary Targets (SDT), Average Requirement (AR) and Reference Intake (RI) are considered. When PRI is not available for a nutrient, Adequate Intake (AI) values are used instead. As any ratio, a NAR equal to 1 indicates that the individual meets the established requirement for that nutrient.

### Adherence to Mediterranean Diet

Adherence to MD is assessed using Mediterranean Diet Adherence Screener (MEDAS), a validated 14-item questionnaire (33). Briefly, one point is attributed for each of the following: (1) olive oil as the main cooking fat; (2) olive oil ≥ 4 tablespoons/day; (3) vegetables ≥ 2 servings/day (≥1 portion raw or on salad); (4) fruit ≥3 servings/day; (5) red or processed meat <1 serving/day; (6) butter or cream or margarine <1/day; (7) sugar-sweetened beverages <1/day; (8) wine ≥3 glasses/week; (9) legumes ≥3 servings/week; (10) fish/seafood ≥3 servings/week; (11) commercial sweets and confectionery <3/week; (12) nuts ≥ 1/week; (13) white more than red meats (yes) and; (14) use of soffritto ≥2/week. A total MEDAS score is calculated by summing the points from all items, obtaining a score ranging from 0 to 14. Subjects with scores above 9 are classified as having high adherence to the MD, those scoring between 6 and 8 have moderate adherence, and scores below 6 indicate low adherence.

### Clinical and neuropsychological assessment

In *phase I,* global cognition is assessed through the Italian version of Montreal Cognitive Assessment Scale (MoCA) screening test, performed by a qualified neuropsychologist blinded to the study.

During *phase II* of the study, patients undergo a more comprehensive neuropsychological assessment. Global cognitive functioning is re-evaluated using the MoCA, while executive functions are assessed with the Frontal Assessment Battery (FAB). Verbal short-term memory is evaluated using the Word Span test, whereas visuospatial short-term memory is assessed with the Corsi Block-Tapping Test. Verbal long-term memory is examined through the Babcock Story Recall Test. Working memory is assessed using the Digit Span Backward test. To evaluate executive-attentional functions, the Attentive Matrices test and the Trail Making Test (TMT), parts A and B, are administered, including an analysis of the difference between the two parts. In addition, phonemic fluency is assessed to investigate language-related aspects and executive functioning. Finally, to further explore the presence of mood disorders, a self-report questionnaire is administered: either the Geriatric Depression Scale (GDS) or the Beck Depression Inventory-II (BDI-II), depending on the patient group or age.

### Data management

In accordance with national and international privacy and anonymization regulations, all these data were stored in an electronic, password-protected database. This database was developed using REDCap (Research Electronic Data Capture), a secure web-based platform designed for database creation and management. Data collection form for *phase I* can be found in the Supplementary materials.

### Ethics statement

Study design has been approved by the local Ethics Committee (Lombardy 4) on November 4, 2024. DIETETICA study is conducted in accordance with the principles outlined in the World Medical Association Declaration of Helsinki. Written informed consent for participation and clinical data collection was obtained from all patients or their legal representatives prior to inclusion. All patient data are handled in compliance with applicable data protection regulations to ensure confidentiality and privacy.

## Statistical data analysis

Preliminary results were obtained using appropriate statistical models based on the type of variable. Descriptive statistics are presented as mean ± standard deviation (M ± SD) for continuous variables and as percentages (%) for categorical variables. The chi-squared test was applied to categorical variables, calculated using GraphPad Prism 8 (GraphPad Software, Inc., San Diego, CA). Continuous variables were analyzed using the Student’s t-test for independent samples. Statistical significance was set at *p* < 0.05 for all tests. *p*-values are represented as follows: * < 0.05; ** < 0.01; *** < 0.001. Missing data were handled using multiple imputation techniques to maintain the integrity of the analyses. The analysis of the 7-dFR was conducted through MetaDieta software (version 4.7.1; *METEDA S.r.l.*).

## Preliminary results

### CADASIL group–risk factors and body composition

We collected 9 CADASIL patients, predominantly female patients (66.66%), with mean age at recruitment of 61 ± 7.16 years. Hypercholesterolemia and hypertriglyceridemia were observed in 33.33 and 22.22% of patients, respectively, while only one patient (11.11%) had hypertension. Smoking history revealed that 33.33% were non-smokers, 44.44% former smokers, and 22.22% current smokers. Alcohol consumption was generally low, with 77.77% consuming alcohol less than twice per week and none exceeding six weekly episodes. No hemorrhagic strokes were recorded in this group. Ischemic strokes and transient ischemic attacks (TIA) were reported in 22.22 and 11.11% of cases, respectively. Cognitive function appeared relatively preserved, with 88.88% of patients classified as non-compromised and only one patient (11.11%) within borderline results. Demographic data, risk factors, and clinical characteristics of the enrolled CADASIL participants are reported in the second column of Table 1.

**Table 1:** Demographic, clinical, and risk factor features of patients enrolled in DIETETICA study.

	Total cohort (*n* = 22)	CADASIL (*n* = 9)	CAA (*n* = 13)	*p*-value
Demographic data				
Male, *n*	15 (68.18)	3 (33.33)	12 (92.30)	0.003**
Female, *n*	7 (31.81)	6 (66.66)	1 (7.69)	0.003**
Age at recruitment, M ± SD (years)	67 ± 9.02	61 ± 7.16	71 ± 8.02	0.006**
Risk factors				
Hypercholesterolemia, *n* (%)	7 (31.81)	3 (33.33)	4 (30.76)	0.898
Hypertriglyceridaemia, *n* (%)	3 (13.63)	2 (22.22)	1 (7.69)	0.328
Hypertension, *n* (%)	8 (36.36)	1 (11.11)	7 (53.84)	0.040 *
Smoking				
Non-smokers, *n* (%)	11 (50)	3 (33.33)	8 (61.53)	0.193
Former smoker, *n* (%)	9 (40.90)	4 (44.44)	5 (38.46)	0.778
Smokers, *n* (%)	2 (9.09)	2 (22.22)	0 (0)	N.A.
Alcohol consumption				
<2 times per week, *n* (%)	15 (68.18)	7 (77.77)	8 (61.53)	0.421
2/6 times per week, *n* (%)	4 (18.18)	2 (22.22)	2 (15.38)	0.682
>6 times per week, *n* (%)	3 (13.63)	0 (0)	3 (23.07)	N.A.
Previous cerebrovascular events				
Hemorrhagic stroke, *n* (%)	10 (45.45)	0 (0)	10 (76.93)	N.A.
Ischemic stroke, *n* (%)	2 (9.09)	2 (22.22)	0 (0)	N.A.
TIA, *n* (%)	3 (13.63)	1 (11.11)	2 (15.38)	0.773
Cognitive function				
Compromised, *n* (%)	4 (18.18)	0 (0)	4 (30.76)	N.A.
Within the limits, *n* (%)	3 (13.63)	1 (11.11)	2 (15.38)	0.773
Non-compromised, *n* (%)	15 (68.18)	8 (88.88)	7 (53.84)	0.082

TIA: transient ischaemic attacks. The two-tailed *t*-test was applied to detect any statistically significant differences between the groups; *p < 0.05; **p < 0.01; N.A.: Not Applicable. Values in bold indicate statistically significant differences (*p* < 0.05).

CADASIL patients presented a mean body weight of 70.8 ± 11.9 kg, body height of 165.8 ± 11.7 cm, and BMI of 25.8 ± 2.5 kg/m^2^. Most patients were of normal weight (55.55%), with 33.33% overweight and one patient (11.11%) classified as obese. Anthropometric circumferences attested a mean arm circumference of 29.1 ± 2.7 cm, waist 97.2 ± 5.8 cm, hip 99.8 ± 6.6 cm, and calf 43.3 ± 19.7 cm. Skinfold thickness measurements were as follows: biceps 10.4 ± 3.5 mm, triceps 17.1 ± 6.0 mm, subscapular 17.5 ± 4.4 mm, and suprailiac 18.8 ± 4.6 mm. FM was 23.9 ± 3.6 kg, while FFM was 46.8 ± 10.6 Kg. Most participants showed low (22.22%) to moderate (33.33%) physical activity levels. Nutritional status was adequate in 66.66%, with no cases of poor nutrition. Regarding adherence to the MD, 66.66% of patients exhibited moderate adherence, while only one patient (11.11%) demonstrated high adherence. Anthropometrical measurements, body composition, and nutritional status of the enrolled CADASIL participants are reported in the second column of Table 2.

**Table 2:** Summary of anthropometric data, body composition parameters, malnutrition risk, and Mediterranean Diet adherence scores of the 22 DIETETICA study participants.

	Total cohort (*n* = 22)	CADASIL (*n* = 9)	CAA (*n* = 13)	*p*-value
Anthropometrics measurements				
BW, M ± SD (kg)	72.9 ± 11.1	70.8 ± 11.9	74.4 ± 10.8	0.480
BH, M ± SD (cm)	169.9 ± 10.0	165.3 ± 11.7	173.1 ± 7.6	0.105
BMI, M ± SD (kg/m^2^)	25.2 ± 2.9	25.8 ± 2.5	24.8 ± 3.9	0.411
Underweight, %	0	0	0	N.A.
Normalweight, *n* (%)	14 (63.63)	5 (55.55)	9 (69.23)	0.592
Overweight, *n* (%)	6 (27.27)	3 (33.33)	3 (23.07)	0.595
Obese, *n* (%)	2 (9.09)	1 (11.11)	1 (7.69)	0.783
Body circumferences				
AC, M ± SD (cm)	28.5 ± 2.7	29.1 ± 2.7	28.1 ± 2.7	0.391
WC, M ± SD (cm)	98.1 ± 8.2	97.2 ± 5.8	98.8 ± 9.7	0.623
HC, M ± SD (cm)	101.4 ± 6.9	99.8 ± 6.6	102.5 ± 7.2	0.368
CC, M ± SD (cm)	38.3 ± 13.2	43.3 ± 19.7	34.8 ± 3.5	0.233
Skinfold thickness				
Biceps, M ± SD (mm)	8.0 ± 3.6	10.4 ± 3.5	6.3 ± 2.7	0.012*
Triceps, M ± SD (mm)	13.3 ± 6.2	17.1 ± 6.0	10.6 ± 5.0	0.018*
Subscapular, M ± SD (mm)	15.4 ± 4.5	17.5 ± 4.4	14.0 ± 4.1	0.073
Suprailiac, M ± SD (mm)	17.0 ± 6.7	18.8 ± 4.6	15.7 ± 7.8	0.262
Body composition				
FM, M ± SD (kg)	21.2 ± 5.3	23.9 ± 3.6	19.3 ± 6.0	0.038*
FFM, M ± SD (kg)	51.7 ± 9.3	46.8 ± 10.6	55.1 ± 6.7	0.062
Physical activity				
<700 METs/week, *n* (%)	12 (54.54)	4 (44.44)	8 (61.56)	0.428
>700 < 2,520 METs/week, *n* (%)	7 (31.81)	3 (33.33)	4 (30.76)	0.898
>2,520 METs/week, *n* (%)	3 (13.63)	2 (22.22)	1 (7.69)	0.328
Nutritional status				
Adequate nutritional status, *n* (%)	13 (59.09)	6 (66.66)	7 (53.84)	0.547
Risk of malnutrition, *n* (%)	8 (36.36)	3 (33.33)	5 (38.46)	0.805
Poor nutritional status, *n* (%)	1 (4.54)	0 (0)	1 (7.69)	N. A.
MEDAS score				
High adherence, *n* (%)	2 (9.09)	1 (11.11)	1 (7.69)	0.783
Moderate adherence, *n* (%)	14 (63.63)	6 (66.66)	8 (61.53)	0.805
Low adherence, *n* (%)	6 (27.27)	2 (22.22)	4 (30.76)	0.132

BW: Body weight; BH: Body height; BMI: Body mass index; AC: Arm circumference; WC: Waist circumference; HC: Hip circumference; CC: Calf circumference; FM: Fat mass; FFM: Fat free mass; METs: metabolic equivalents; MEDAS: Mediterranean Diet Adherence Screener. The two-tailed *t*-test was applied to detect any statistically significant differences between the groups; *p < 0.05; **p < 0.01; N.A.: Not Applicable. Values in bold indicate statistically significant differences (*p* < 0.05).

### CAA group–risk factors and body composition

The CAA patient group (*n* = 13) had a mean age at recruitment of 71 ± 8.02 years, and the majority of patients were male (92.30%). The most prevalent vascular risk factor was hypertension, present in 53.84% of cases, followed by hypercholesterolemia (30.76%) and hypertriglyceridemia (7.69%). No active smokers were reported, whereas 38.46% were former smokers and 61.53% had never smoked. Regarding alcohol consumption, 61.53% reported drinking less than twice per week, while 15.38% drank 2–6 times weekly and 23.07% consumed alcohol more than 6 times per week. Hemorrhagic stroke was the most frequent cerebrovascular event, affecting 76.93% of patients, while no Ischemic strokes were reported. Transient Ischemic attacks (TIA) occurred in 15.38% of cases. Cognitive function was classified as compromised in 30.76%, impaired but with borderline results in 15.38%, and non-compromised in 53.84% of patients. Demographic data, risk factors, and clinical characteristics of the enrolled CAA patients are reported in the third column of Table 1.

Cerebral amyloid angiopathy patients had a mean body weight of 74.4 ± 10.8 kg, body height of 173.1 ± 7.6 cm, and a mean BMI of 24.8 ± 3.9 kg/m^2^. Based on BMI classification, 69.23% of participants were of normal weight, 23.07% overweight, and 7.69% obese. Body circumference measurements revealed mean values of 28.1 ± 2.7 cm for the arm, 98.8 ± 9.7 cm for the waist, 102.5 ± 7.2 cm for the hip, and 34.8 ± 3.5 cm for the calf. Skinfold thickness values were 6.3 ± 2.7 mm at the biceps, 10.6 ± 5.0 mm at the triceps, 14.0 ± 4.1 mm at the subscapular site, and 15.7 ± 7.8 mm at the suprailiac site. FM averaged 19.3 ± 6.0 kg, while FFM was 51.1 ± 6.7 kg. Physical activity levels were predominantly low, with 61.56% of participants reporting less than 700 METs/week, while only one patient exceeded 2,520 METs/week. Nutritional status was adequate in most of patients (53.84%), whereas 38.46% were at risk of malnutrition, and one patient showed poor nutritional status. According to the MEDAS score, the majority of participants (61.53%) demonstrated moderate adherence to MD, while only one patient (7.69%) showed high adherence to this dietary pattern. Anthropometrical measurements, body composition, and nutritional status of the enrolled CAA participants are reported in the third column of Table 2.

### Description of daily dietary nutrient intake and nutritional adequacy ratio (NAR)

Table 3 shows the mean and standard deviation (M ± SD) of average daily intakes of energy, protein, available carbohydrates, fiber and total fats, as well as minerals (calcium, potassium, iron, phosphorus, sodium) and vitamins (A, D, E, C, B9, B12), for the whole study cohort. No statistically significant differences were observed between CADASIL and CAA patients in terms of daily energy intake and distribution across macronutrients and micronutrients.

**Table 3:** Median daily food intake from 7-dFR of the 22 participants enrolled in DIETETICA study.

	Total cohort (*n* = 22)	CADASIL (*n* = 9)	CAA (*n* = 13)	*p*-value
Macronutrient				
Energy, M ± SD, (kcal/d)	1781.1 ± 162.4	1724.6 ± 116.4	1820.2 ± 181.9	0.148
Protein, M ± SD, (g/d)	67.9 ± 10.5	70.4 ± 8.2	66.2 ± 11.9	0.336
Available carbohydrates, M ± SD, (g/d)	228.9 ± 39.5	220.3 ± 45.6	234.8 ± 35.4	0.437
Fiber, M ± SD, (g/d)	18.2 ± 3.8	17.5 ± 5.0	18.7 ± 2.8	0.538
Total fats, M ± SD, (g/d)	66.7 ± 10.3	63.9 ± 9.2	68.7 ± 11.0	0.289
SFAs, M ± SD, (g/d)	20.6 ± 4.2	21.2 ± 4.0	20.2 ± 3.8	0.583
MUFAs, M ± SD, (g/d)	27.8 ± 10.0	23.8 ± 4.7	30.6 ± 11.6	0.076
PUFAs, M ± SD, (g/d)	7.9 ± 3.5	6.9 ± 2.1	8.6 ± 4.1	0.223
PUFAs ω-6, M ± SD, (g/d)	4.2 ± 2.5	4.1 ± 1.7	4.3 ± 3.0	0.815
PUFAs *ω*-3, M ± SD, (g/d)	0.7 ± 0.5	0.7 ± 0.3	0.8 ± 0.6	0.629
EPA, M ± SD, (mg/d)	62.5 ± 56.0	71.1 ± 63.1	56.5 ± 52.4	0.577
DHA, M ± SD, (mg/d)	86.7 ± 77.6	83.3 ± 86.0	89.1 ± 74.8	0.871
Minerals				
*Ca,* M ± SD, (mg/d)	490.9 ± 138.7	500.1 ± 185.1	484.5 ± 108.1	0.822
K, M ± SD, (mg/d)	2.3 ± 0.5	2.1 ± 0.4	2.4 ± 0.5	0.787
Fe, M ± SD, (mg/d)	7.7 ± 1.5	7.0 ± 1.3	8.2 ± 1.5	0.366
P, M ± SD, (mg/d)	802.0 ± 154.8	791.2 ± 144.8	809.5 ± 174.2	0.337
Na, M ± SD, (mg/d)	1.4 ± 0.3	1.5 ± 0.4	1.3 ± 0.3	0.068
Vitamins				
Vit A, M ± SD, (μg/d)	1285.5 ± 666.4	1141.1 ± 391.7	1385.5 ± 805.3	0.357
Vit. D, M ± SD, (μg/d)	1.1 ± 0.6	0.8 ± 0.2	1.3 ± 0.7	0.514
Vit. E, M ± SD, (mg/d)	5.2 ± 1.2	4.9 ± 1.9	5.4 ± 0.6	0.066
Vit. C, M ± SD, (mg/d)	85.0 ± 30.5	73.5 ± 29.7	93.0 ± 29.5	0.146
Vit. B9, M ± SD, (μg/d)	210. 0 ± 54.9	190.7 ± 39.0	223.4 ± 61.6	0.779
Vit. B12, M ± SD, (μg/d)	3.1 ± 3.3	2.9 ± 2.8	3.3 ± 3.7	0.143

d: die; SFAs: saturated fatty acids; MUFAs: monounsaturated fatty acids; PUFAs: polyunsaturated fatty acids; EPA: eicosapentaenoic acid; DHA: docosahexaenoic acid; Ca: calcium; K: potassium; Fe: iron; P: phosphorus; Na: sodium; g: grams; mg: milligram; μg: microgram; Vit: vitamin. The two-tailed *t*-test was applied to detect any statistically significant differences between the groups; *p* < 0.05.

### CADASIL group—nutritional adequacy ratio

In CADASIL patients, daily energy intake (kcal/d) and fiber intake (%En) were inadequate, with median NARs of 0.80 and 0.86, respectively. Conversely, protein intake (g/kg x d) and available carbohydrate intake (%En) exceeded DRVs. The intake of total fats (%En), SFAs (%En), and MUFAs (%En) was notably higher than recommended levels, as reflected by median NARs of 1.55, 1.41, and 1.56, respectively. However, none of the patients reached the DRVs for total PUFAs (%En), PUFAs *ω*-6 and ω-3, EPA (mg/d), and DHA (mg/d).

Approximately 70 and 60% of CADASIL patients had phosphorus (mg/d) and sodium (g/d) intakes exceeding DRVs, with median NARs of 1.13 and 1.23, respectively. In contrast, none of the patients achieved recommended intake levels for calcium (mg/d), potassium (g/d), and iron (mg/d). Significantly higher median NARs of 1.82 and 1.22 were observed for vitamin A (μg/d) and vitamin B12 (μg/d), respectively, indicating an intake above the recommended levels for these nutrients. In contrast, intakes of vitamin D (μg/d), vitamin E (mg/d), and vitamin B9 (μg/d) were below DRVs. Table 4 shows the proportion of participants with a nutrient intake above, below, or within the recommendations, together with the corresponding DVRs and median NARs.

**Table 4:** Estimated NARs for macronutrients and micronutrients of 22 patients enrolled in DIETETICA study.

	DRVs	Total cohort (*n* = 22)		CADASIL (*n* = 9)		CAA (*n* = 13)	
		Patient with “adeguate” intake n (%)	NAR (median)	Patient with “adeguate” intake n (%)	NAR (median)	Patient with “adeguate” intake n (%)	NAR (median)
Macronutrients							
Energy, (kcal/d)	AR	2 (9.09)	0.83	2 (11.1)	0.80	0 (0)	0.88
Protein, (g/kg × d)	STD / PRI	2 (9.09)	0.92	1 (11.11)	1.03	1 (7.69)	0.84
Available carbohydrates, (%En)	RI	18 (81.81)	1.15	7 (77.77)	1.13	11 (84.61)	1.15
Fiber, (%En)	RI	5 (22.72)	0.87	3 (33.33)	0.86	2 (15.38)	0.88
Total fats, (%En)	RI	21 (90.9)	1.56	2 (11.11)	1.55	2 (15.38)	1.56
SFAs, (%En)	STD	18 (81.81)	1.32	7 (77.77)	1.41	11 (84.61)	1.26
MUFAs, (%En)	RI^1^	21 (95.45)	1.77	8 (88.87)	1.56	13 (100)	1.92
PUFAs, (%En)	RI	3 (13.63)	0.67	0 (0)	0.60	3 (23.0)	0.71
PUFAs ω-6, (%En)	RI	2 (9.09)	0.45	0 (0)	0.44	2 (15.38)	0.46
PUFAs ω-3, (%En)	RI	1 (4.54)	0.34	1 (0)	0.32	1 (7.69)	0.35
EPA, (mg/d)	AI	0 (0)	0.25	0 (0)	0.28	0 (0)	0.22
DHA, (mg/d)	AI	1 (4.54)	0.34	0 (0)	0.33	1 (7.69)	0.35
Minerals							
*Ca,* (mg/d)	PRI	0 (0)	0.42	0 (0)	0.43	0 (0)	0.41
K, (g/d)	AI	0 (0)	0.59	0 (0)	0.56	0 (0)	0.61
Fe, (mg/d)	PRI	2 (9.09)	0.77	0 (0)	0.70	2 (15.38)	0.82
P, (mg/d)	PRI	18 (81.81)	1.14	7 (77.77)	1.13	11 (84.61)	1.15
Na, (g/d)	AI	12 (54.54)	1.16	6 (66.66)	1.23	6 (46.15)	1.11
Vitamins							
Vit. A, (μg/d)	PRI	21 (95.45)	1.93	9 (100)	1.82	12 (92.30)	2.01
Vit. D, (μg/d)	PRI	0 (0)	0.07	0 (0)	0.05	0 (0)	0.08
Vit. E, (mg/d)	PRI	0 (0)	0.41	0 (0)	0.40	0 (0)	0.41
Vit. C, (mg/d)	PRI	7 (31.81)	0.86	3 (33.33)	0.82	4 (30.76)	0.89
Vit. B9, (μg/d)	PRI	0 (0)	0.52	0 (0)	0.47	0 (0)	0.55
Vit. B12, (μg/d)	PRI	9 (40.90)	1.31	4 (44.44)	1.22	5 (38.46)	1.38

SFAs: saturated fatty acids; MUFAs: monounsaturated fatty acids; PUFAs: polyunsaturated fatty acids; EPA: eicosapentaenoic acid; DHA: docosahexaenoic acid; Ca: calcium; K: potassium; Fe: iron; P: phosphorus; Na: sodium; d: die; g: grams; mg: milligram; μg: microgram; Vit: vitamin; En: energy; DRVs: Dietary Reference Values based on the Italian LARN, IV Revision; AR: average requirement; RI: Reference Intake; STD: suggested dietary targets; AI: adequate intake; 1 Reference Intake calculated by difference: RI (MUFAs) = RI (total fats) – RI (PUFAs) – STD (SFAs).

### CAA group—nutritional adequacy ratio

CAA patients demonstrated inadequate daily energy intake (kcal/d), as highlighting by a median NAR of 0.88. Additionally, protein (g/kg x d) and fiber (%En) intake were below the DRVs, with only 7.69 and 15.38% of patients meeting the adequate intake for these nutrients, respectively. Conversely, the intake of available carbohydrates (%En) and total fats (%En) exceeded the RI upper limit, with median NARs of 1.15 and 1.56. Approximately, 84% of CAA patient exceeding the STD cut-off value for SFAs (%En), as reflected by a median NAR bigger than 1. Despite total fats intakes being higher than recommended levels, the intakes of PUFAs *ω*-3 (%En), including its derivatives EPA (mg/d) and DHA (mg/d), and PUFAs ω-6 (%En), did not reach the DRVs. Furthermore, significantly higher intakes were observed for MUFAs (%En), with a median NAR of 1.92.

For calcium (mg/d), potassium (mg/d), and iron (mg/d), the median NARs were 0.41, 0.61, and 0.82, respectively, indicating significant deficits intakes of those nutrients. In contrast, optimal intakes of phosphorus and sodium were observed in CAA patients, as reflected by median NARs of 1.15 and 1.11, respectively. Lastly, significantly higher intakes were recorded for vitamin A (μg/d) and vitamin B12 (μg/d), while median NARs for vitamin D (μg/d), vitamin E (mg/g), vitamin C (mg/d) and vitamin B9 (μg/d) were below 1, indicating a notable inadequacy in the intake of these nutrients.

Table 4 shows the proportion of participants with a nutrient intake above, below, or within the recommendations, together with the corresponding DVRs and median NARs.

Collectively, these findings suggest a dietary pattern characterized by a low consumption of key plant-based foods such as legumes, nuts, and whole grains (leading to low fiber and PUFA intake), and certain fruits and vegetables (source of potassium and vitamins). The inadequate intake of calcium and vitamin D may reflect a low consumption of dairy products or fortified foods. Conversely, the excessive intake of sodium and saturated fats likely points to a high consumption of processed foods, red meat, and certain cheeses.

## Discussion

DIETETICA is the first study aimed at evaluating anthropometric and body composition parameters, as well as investigating the effect of a MD on stroke incidence and cognitive impairment in patients with CADASIL and CAA. It is known that these two different causes of cSVD, hereditary and acquired respectively, share some common clinical and molecular features; however, their distinct pathophysiology and clinical progression may influence body composition as well as the response to usual dietary patterns.

In our preliminary results, patients with CADASIL were younger (mean age 61 ± 7.16 years), predominantly female (66.7%), and showed relatively preserved cognitive function and higher FM in comparison to CAA. Conversely the CAA patients were older individuals (mean age 71 ± 8.02 years), mostly male (92.3%), with a greater prevalence of hypertension, hemorrhagic strokes, and of cognitive impairment. No other differences were found between the demographic factors of the two diseases.

Body composition assessment showed that bicipital and tricipital skinfold thicknesses, as well as FM (Kg), differed significantly between the CADASIL and CAA groups. These differences in body composition could reflect the different pathophysiological profiles and long-term outcomes associated with distinct stroke subtypes. Supporting this hypothesis, Wilczyński and colleagues (34) demonstrated that individuals recovering from a hemorrhagic stroke have significantly reduced FM, compared to survivors of an ischemic stroke, which the authors attributed to a combination of more severe neurological damage, higher metabolic demands and complications, such as dysphagia. Our results are concordant with these findings: patients with CAA, most of whom experienced hemorrhagic strokes, presented lower FM and reduced skinfold measurements, when compared to CADASIL patient. However, these results should be interpreted carefully, given the unbalanced nature of our sample, which could limit the generalizability of the findings.

The overall low adherence to the Mediterranean Diet (MD) in our cohort, with only 9% of participants achieving a high MEDAS score, is a robust finding supported by both internal and external evidence. Internally, this score is not merely a subjective result from a questionnaire but is quantitatively confirmed by our nutritional adequacy analysis, which revealed a dietary pattern deficient in classic Mediterranean components like polyunsaturated fatty acids (PUFAs) while being excessive in saturated fats and sodium. Externally, this finding is consistent with broader dietary trends in Northern Italy, as a similar study (24) on free-living elderly individuals in the region also reported a low rate of high adherence (approximately 30%). Taken together, this suggests that the dietary habits of our patient cohort reflect a wider regional pattern that is quantitatively suboptimal. In a recent systematic review, analyzing adherence among adults in Mediterranean countries, only 20–45% of individuals were found to achieve moderate to high levels of adherence to MD, with a decreasing trend across age groups and a notable deterioration in the last decades (35). Our hypothesis that the Mediterranean Diet may offer neuroprotection in cSVD is supported by evidence from the general population. A study on elderly urban Italians found that closer adherence to the MD was significantly associated with a better cognitive status, reducing the risk of cognitive impairment by over 60% (24). Our study extends this line of inquiry by investigating whether this protective association holds true in a genetically and pathologically defined high-risk population. Our findings emphasize the need for targeted dietary interventions to increase adherence to this traditional pattern, which has been shown to reduce the incidence and mortality from cerebrovascular disease (36). Furthermore, the high prevalence of physical inactivity observed, highlights the necessity of comprehensive lifestyle modifications, especially because it is well-known that regular physical activity is a key factor in maintaining cognitive function and preventing neurodegenerative processes (37).

To our knowledge, no study has yet examined the average daily nutrient intake in patients with cSVD. The data presented in Table 3 provide a valuable reference for cross-sectional comparisons, facilitating the identification of differences and similarities in dietary patterns. In addition, analysis of these intake levels could help to explore associations between nutrient consumption and cerebrovascular outcomes or cognitive impairment. A central finding of our nutritional analysis is the profound imbalance in lipid quality, which is more relevant than the total fat quantity. The excessive intake of SFAs, known for their pro-inflammatory and atherogenic potential (38, 39), combined with a striking deficit of anti-inflammatory and neuroprotective PUFAs (*ω*-3, EPA, DHA) (40–43), delineates a dietary profile that may actively contribute to the vascular and neuro-inflammatory processes underlying cSVD. This highlights that future dietary interventions must prioritize fat quality, promoting sources of unsaturated fats like olive oil, nuts, and fish, while actively reducing saturated fat intake.

While our findings might provide novel insights, they are limited by the small sample size and unbalanced sex distribution between diagnostic groups. Additionally, the monocentric and hospital-based design of the study may introduce a selection bias and limit the generalizability of the results to the broader population. Moreover, body composition was estimated using skinfold thickness measurements, rather than directly measured with the gold-standard dual-energy X-ray absorptiometry (DEXA) method, potentially affecting the precision of FM and FFM assessments. Regarding the assessment of Mediterranean Diet adherence, the MEDAS questionnaire, although a validated and widely used screener, has inherent limitations. Its semi-quantitative nature and scoring thresholds may not fully capture the nuances of dietary quality; for instance, the requirement of consuming nuts ‘≥1 time/week’ to score a point is a low threshold for a diet where nuts are a key component. This could lead to an overestimation of adherence levels in individuals with only marginal consumption. Furthermore, concerning the quantitative nutritional assessment, our analysis relies on the 7-day food record, which is the gold standard for intake evaluation. However, the accuracy of nutrient calculations is inherently dependent on the completeness of the food composition databases within the analysis software. While MetaDieta is a comprehensive tool, information for specific fatty acid profiles—such as individual monounsaturated (MUFAs) and polyunsaturated (PUFAs) fatty acids—is often not reported on commercial food labels and can be lacking in databases. This may have led to an underestimation of the intake of these specific nutrients, despite our use of the best available methodology. These limitations, however, do not detract from our preliminary findings, which highlight significant trends in dietary patterns that warrant further investigation in Phase II of our study.

Nonetheless, a strength of this study is that the dietary records used, based on a 7-dFR, represent the gold standard for providing accurate quantification of daily food intake and allowed accurate assessment of nutritional adequacy. Therefore, the findings from this observational phase are not merely descriptive but serve as a crucial foundation for Phase II. The planned intervention, which focuses on supplementing the Mediterranean Diet with olive oil or dried fruit, is specifically designed to address the key nutritional gaps identified herein—particularly the inadequate intake of MUFAs and PUFAs. Phase II will thus directly test whether correcting these specific, observed dietary deficits can impact stroke incidence and cognitive decline in patients with CADASIL and CAA, moving from observation to targeted, evidence-based intervention.

## Data Availability

The original contributions presented in the study are included in the article/Supplementary material, further inquiries can be directed to the corresponding author/s.
