# Supplementary material for: Impact of the Mediterranean Diet on stroke incidence and cognitive impairment in CADASIL and CAA patients: the DIETETICA study
Source: Front Nutr. 2025 Nov 25;12:1682134. doi: 10.3389/fnut.2025.1682134 (PMC12685673; doi:10.3389/fnut.2025.1682134)
Supplement: Supplementary file 1 [file Data_Sheet_1.PDF]

DATA COLLECTION FOR PHASE I

Demographic data

Sex

Date of Birth

Place of Birth

Date of Enrollment

Age at Enrollment

Risk factors

Smoking status

Alcohol consumption

Presence of hypercholesterolemia

Presence of hypertriglyceridemia

Presence of diabetes

Presence of hypertension

Atrial fibrillation

Previous cerebrovascular events

Heamhorragic stroke

Ischemic stroke

Transient ischaemic attacks

Date of all cerebrovascular events

Anthropometrics measurements

Body weight

Body height

Body mass index

Body circumference (arm circumference, waist circumference, hip circumference and calf circumference)

Waist-to-hip ratio

Skinfold thickness (biceps, triceps, subscapular and suprailiac)

Body composition (Fat Mass and Fat Free Mass)

Estimate of basal metabolic rate (BMR)

Estimate of Total energy expenditure (TEE)

Physical activity level

METs derived from the International Physical Activity Questionnaire (IPAQ) questionnaire

Physical activity level based on the World Health Organization (WHO) guidelines

Cognitive functions

Corrected score of the MoCA screening test

Raw score of the MoCA screening test

Nutritional status

Score derived from the Mini Nutritional Assessment (MNA) questionnaire

Adherence to Mediterranean Diet

Score derived from the Mediterranean Diet Adherence Screener (MEDAS) questionnaire

Nutritional assessment - Estimated average daily intake over a 7-day period

Kilocalories

Percentage of daily energy intake from carbohydrates

Grams of carbohydrates consumed per day

Grams of fiber consumed per day

Percentage of daily energy intake from proteins

Grams of protein consumed per day

Percentage of daily energy intake from lipids

Grams of saturated fatty acids consumed per day

Grams of polyunsaturated fatty acids consumed per day

Grams of monounsaturated fatty acids consumed per day

Grams of omega-6 polyunsaturated fatty acids consumed per day

Grams of omega-3 polyunsaturated fatty acids consumed per day

Milligrams of calcium consumed per day

Grams of potassium consumed per day

Milligrams of sodium consumed per day

Milligrams of iron consumed per day

Milligrams of magnesium consumed per day

Milligrams of zinc consumed per day

Micrograms of Vitamin B12 consumed per day

Micrograms of Vitamin B6 consumed per day

Milligrams of Vitamin B3 consumed per day

Milligrams of Vitamin C consumed per day

Micrograms of Vitamin A consumed per day

Micrograms of Vitamin D consumed per day

Micrograms of Vitamin E consumed per day

Laboratory test results

Total cholesterol

LDL cholesterol

HDL cholesterol

Triglycerides

Blood glucose

Glycated haemoglobin (HbA1c)

C-reactive protein (CRP)

Aspartate aminotransferase (AST)

Alanine aminotransferase (ALT)

Gamma-glutamyl transferase (Gamma-GT)

Thyroid-stimulating hormone (TSH)

Serum creatinine

Serum sodium

Serum potassium

Calcium

Homocysteine

Vitamin B12

Vitamin D

Folate

Iron

Ferritin
